# Development of a Novel Vital-Signs-Based Infection Screening Composite-Type Camera With Truncus Motion Removal Algorithm to Detect COVID-19 Within 10 Seconds and Its Clinical Validation

**DOI:** 10.3389/fphys.2022.905931

**Published:** 2022-06-22

**Authors:** Batbayar Unursaikhan, Gereltuya Amarsanaa, Guanghao Sun, Kenichi Hashimoto, Otgonbat Purevsuren, Lodoiravsal Choimaa, Takemi Matsui

**Affiliations:** ^1^ Graduate School of Systems Design, Tokyo Metropolitan University, Hachioji, Japan; ^2^ Machine Intelligence Laboratory, School of Engineering and Applied Sciences, National University of Mongolia, Ulaanbaatar, Mongolia; ^3^ The First Central Hospital of Mongolia, Ulaanbaatar, Mongolia; ^4^ Graduate School of Informatics and Engineering, The University of Electro-Communications, Chofu, Japan; ^5^ Department of General Medicine, National Defense Medical College, Tokorozawa, Japan

**Keywords:** infection screening, COVID-19, non-contact vital signs measurement, mobile screening system, remote photoplethysmograph, body temperature measurement, respiratory rate measurement, heart rate measurement

## Abstract

**Background:** To conduct a rapid preliminary COVID-19 screening prior to polymerase chain reaction (PCR) test under clinical settings, including patient’s body moving conditions in a non-contact manner, we developed a mobile and vital-signs-based infection screening composite-type camera (VISC-Camera) with truncus motion removal algorithm (TMRA) to screen for possibly infected patients.

**Methods:** The VISC-Camera incorporates a stereo depth camera for respiratory rate (RR) determination, a red–green–blue (RGB) camera for heart rate (HR) estimation, and a thermal camera for body temperature (BT) measurement. In addition to the body motion removal algorithm based on the region of interest (ROI) tracking for RR, HR, and BT determination, we adopted TMRA for RR estimation. TMRA is a reduction algorithm of RR count error induced by truncus non-respiratory front-back motion measured using depth-camera-determined neck movement. The VISC-Camera is designed for mobile use and is compact (22 cm × 14 cm × 4 cm), light (800 g), and can be used in continuous operation for over 100 patients with a single battery charge. The VISC-Camera discriminates infected patients from healthy people using a logistic regression algorithm using RR, HR, and BT as explanatory variables. Results are available within 10 s, including imaging and processing time. Clinical testing was conducted on 154 PCR positive COVID-19 inpatients (aged 18–81 years; M/F = 87/67) within the initial 48 h of hospitalization at the First Central Hospital of Mongolia and 147 healthy volunteers (aged 18–85 years, M/F = 70/77). All patients were on treatment with antivirals and had body temperatures <37.5°C. RR measured by visual counting, pulsimeter-determined HR, and BT determined by thermometer were used for references.

**Result:** 10-fold cross-validation revealed 91% sensitivity and 90% specificity with an area under receiver operating characteristic curve of 0.97. The VISC-Camera-determined HR, RR, and BT correlated significantly with those measured using references (RR: *r* = 0.93, *p* < 0.001; HR: *r* = 0.97, *p* < 0.001; BT: *r* = 0.72, *p* < 0.001).

**Conclusion:** Under clinical settings with body motion, the VISC-Camera with TMRA appears promising for the preliminary screening of potential COVID-19 infection for afebrile patients with the possibility of misdiagnosis as asymptomatic.

## Introduction

As of March 2022, the number of people who have been infected with COVID-19 worldwide was 434 million, resulting in approximately 5.9 million deaths (WHO, 2022). Although nucleic acid amplification tests, such as the reverse transcriptase-polymerase chain reaction (RT-PCR), are recommended to identify active infection as a gold standard, the tests require specialty resources and time-intensive tasks (WHO, 2020a). RT-PCR test takes approximately 2 h, including specimen collection and handling time.

As a preliminary daily screening prior to the RT-PCT test, an infrared thermometer-based body temperature (BT) screening has been widely used to conduct rapid infection screening at entrances of mass gathering places such as schools, offices, and hospitals (WHO, 2020b). However, recent cohort studies have reported a high rate (70–75%) of afebrile cases in patients with COVID-19 infection, which will be undetected in BT screening and can spread the virus (Goyal et al., 2020; Richardson et al., 2020). Rechtman et al. have reported COVID-19 induced vital signs alterations in addition to BT, such as higher heart rate (HR, HR>100 bpm) than that of normal controls and higher respiratory rate (RR, RR>24 bpm) (Rechtman et al., 2020). Moreover, a cohort study in the United Kingdom revealed that patients with COVID-19 experienced significant increases in RR and slight increases in HR; however, they had no significant increases in BT (Pimentel et al., 2020).

**FIGURE 1:**
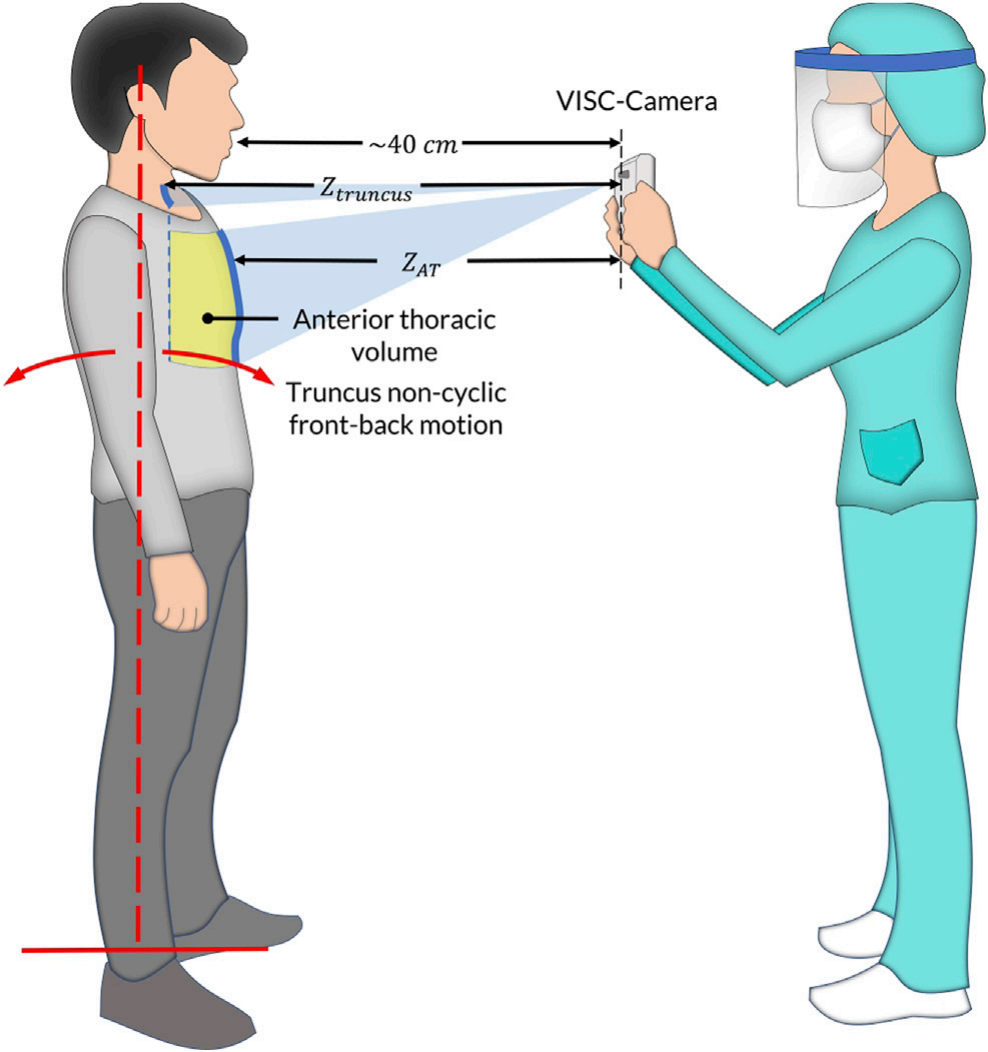
The vital-signs-based infection screening composite-type camera (VISC-Camera) measurement setup in the standing position. In addition to respiratory anterior thorax ( $Z_{AT}$ ) region of interest (ROI) tracking algorithms, the VISC-Camera incorporates a non-respiratory truncus motion (red dotted line) removal algorithm (TMRA) using $Z_{truncus\;}$ movements determined as neck motions.

We have developed vital signs (RR, HR, and BT) based infection screening system which can detect afebrile patients (Matsui et al., 2010; Dagdanpurev et al., 2019). However, the proposed system had a limitation of accuracy in orthostatic RR measurement because of the patient’s non-respiratory motion, such as truncus front-back motion, for maintaining upright posture (Figure 1). Using Doppler radars, we have previously developed non-contact screening systems for sepsis and pneumonia (Matsui et al., 2020; Otake et al., 2021). However, Doppler radars have limitations in noise reduction induced by body movements. Therefore, in this paper, we adopted image sensors used in our previous studies instead of Doppler radars, such as a depth camera (Takamoto et al., 2020) and a red–green–blue (RGB) camera (Unursaikhan et al., 2021).

**FIGURE 2:**
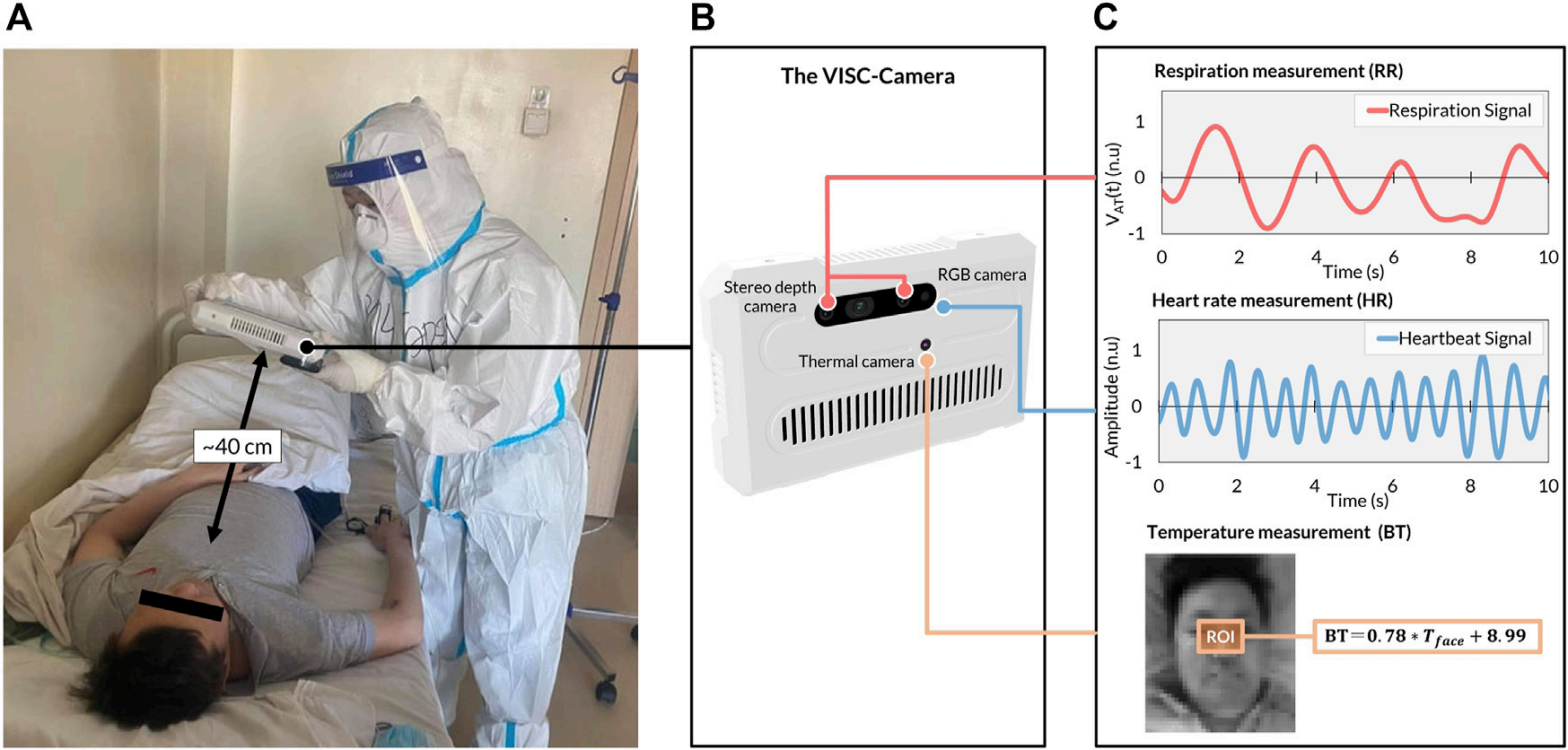
The VISC-Camera structure and vital signs-related signals were derived in the COVID-19 isolation unit. **(A)** VISC-Camera screening for a patient with COVID-19 within 10 s **(B)** The three cameras of VISC-Camera: stereo depth camera for respiratory rate (RR) monitoring, RGB camera for heart rate (HR) determination, thermal camera for body temperature (BT) measurement. **(C)** Vital signs-related signals of the patient comprising respiration signal, heartbeat signal, and facial region of interest (ROI, orange rectangle) for BT determination.

In order to conduct an accurate orthostatic RR assessment, we propose a truncus motion removal algorithm (TMRA). A truncus motion is measured using depth-camera-determined neck movement. Adopting TMRA in addition to the ordinary region of interest (ROI) tracking algorithms, we developed a mobile and vital-signs-based infection screening composite-type camera (VISC-Camera). VISC-Camera detects COVID-19 possibly infected patients within 10 s using a stereo depth camera for RR determination, an RGB camera for HR estimation, and a thermal camera for BT measurement (Figure 2). These three vital signs are indices used to determine the systemic inflammatory response syndrome (SIRS) score (Kaukonen et al., 2015).

To conduct a rapid preliminary COVID-19 screening, we developed a portable vital-signs based COVID-19 screening system using multiple image sensors (VISC-Camera) with body (trunk) motion removal algorithm (TMRA). The clinical study in a Mongolian hospital revealed that VISC-camera enabled accurate afebrile COVID-19 patients screening due to patients’ respiratory rate (RR) increase induced by inflammatory responses in relation to COVID-19. Clinical testing was conducted on 154 PCR positive COVID-19 inpatients (aged 18–81 years; M/F = 87/67) within the initial 48 h of hospitalization at the First Central Hospital of Mongolia and 147 healthy volunteers (aged 18–85 years, M/F = 70/77).

## Materials and Methods

### The VISC-Camera System Structure

The VISC-Camera with a seven-inch touchscreen display incorporates three kinds of image sensors, i.e., a stereo depth camera with a Red-Green-Blue (RGB) camera (424 × 240 pixels, 30 [frames/s], Intel Real sense D435i), and a thermal camera [80 × 60 pixels, 9 (frames/s), FLIR Lepton 2.5] (Figure 2B). A circular polarizer lens is installed in front of the RGB camera to reduce the reflection of glossy skin. The VISC-Camera has a built-in single-board computer (Raspberry Pi 4B) for data processing. Images of a stereo depth camera with an RGB camera and a thermal camera are transferred to the single board computer through USB 3.0 and serial peripheral interface (SPI), respectively. A built-in Li-ion battery enables continuous operation for over 100 measurements by a single battery charge. The housing of the device is printed by a 3D printer with overall dimensions of 22 cm (L) × 14 cm (W) × 4 cm (H). The total weight of the VISC-Camera is 800 g.

### The VISC-Camera Data Processing Algorithms

**FIGURE 3:**
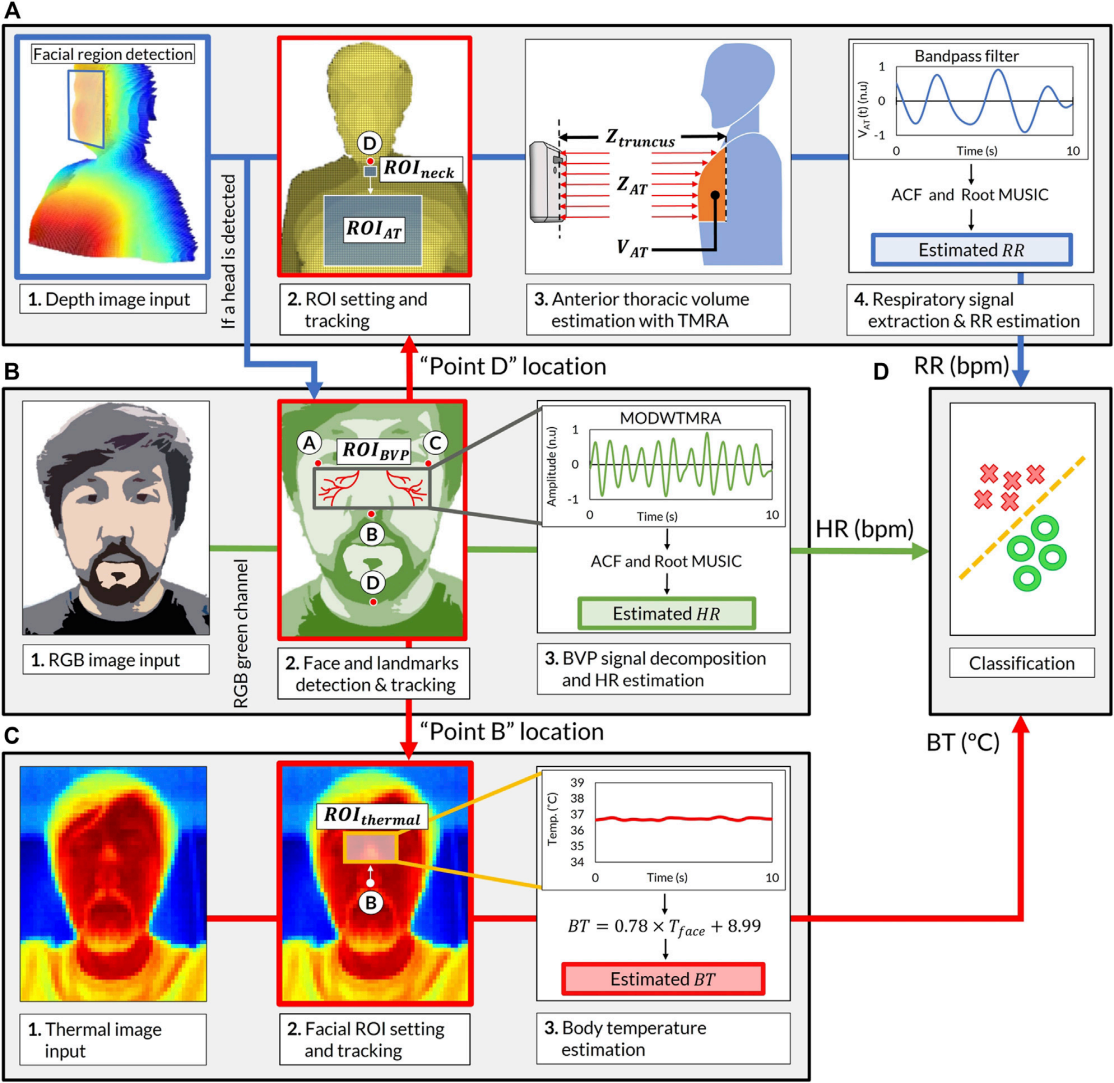
Software block diagram of the VISC-Camera screening procedure. **(A)** Respiratory rate (RR) estimation procedure. (Chart 1) Stereo depth camera capture [424 × 240 pixels, 30 (frames/s)]. (Chart 2) $ROI_{neck}$ (3 × 3 cm) for truncus-motion monitoring is placed below point D. $ROI_{AT}$ [20 (H) cm ×18 (W) cm] for anterior thorax measurement is fixed 12 cm below the D. (Chart 3) Estimation of anterior thoracic volume, $V_{AT}\left(t\right)$ , using truncus motion removal algorithm (TMRA), which separates non-respiratory truncus-motion [ $Z_{truncus}\left(t\right)$ ] from respiratory induced anterior thorax-motion [ $Z_{AT}\left(t\right)$ ]. (Chart 4) Extraction of the respiratory signal using autocorrelation function (ACF) and root MUSIC algorithm. **(B)** Heart rate (HR) estimation procedure. (Chart 1) RGB camera capture [424 × 240 pixels, 30 (frames/s)]. (Chart 2) $ROI_{BVP}$ for HR monitoring is determined by facial landmarks A-C. (Chart 3) Heartbeat signal obtained by wavelet transformation (MODWTMRA) and root MUSIC algorithm. **(C)** Body temperature (BT) estimation procedure. (Chart 1) Thermal camera capture (80 × 60 pixels, 9 [frames/s]). (Chart 2) $ROI_{thermal\;}$ (5 × 7 cm) is placed 3 cm above the landmark point B. (Chart 3) BT determined by the estimated equation from facial temperature. **(D)** Classification of patients with COVID-19 infections from healthy volunteers using explanatory variables of RR, HR, and BT via logistic regression analysis. ROI, region of interest; MUSIC, multiple signal classification; BVP, blood volume pulse; MODWTMRA, multiresolution analysis of the maximal overlap discrete wavelet transform.

The software of the VISC-Camera is stand-alone and was developed in Python programming language (Python Software Foundation). The overview of the software block diagram is shown in Figure 3 (Supplementary Video). We used the Open computer vision (OpenCV) library for image processing and the multiprocessing module from Python to execute the image analyses simultaneously (Bradski, 2000). The VISC-Camera executes parallel processes in a quad-core processor, i.e., image capturing and RR, HR, and BT determinations. As shown in *The Truncus Motion Removal Algorithm for Respiratory Rate Estimation*, the VISC-Camera extracts RR from depth images while measuring the distance from the VISC-camera to the examinee. RGB images enable HR estimation through facial landmarks detection with a facial tracking algorithm. BT is derived from RGB-determined ROI using the thermal images. Logistic regression analysis (LRA) was adopted from the Python Scikit-Learn machine-learning library (Pedregosa et al., 2011) to classify COVID-19 infection. The linear equation determined by LRA is expressed as follows:

$$\begin{array}{c} \log\frac{p}{\mathrm{1-p}}=\beta_{0}+\beta_{1}\times RR+\beta_{2}\times HR+\beta_{3}\mathrm{\times BT} \end{array}$$ (1)

where $\log\frac{p}{1-p}\;$ is the predicted logit score, $\beta_{0}\;$ is a constant, and $\beta_{1}\cdots \beta_{3}\;$ are regression coefficients corresponding to the LRA explanatory variables of RR, HR, and BT (Figure 3D).

In order to keep the computational load as minimum as possible, we adopted the following procedures to determine regions of interest. $ROI_{BVP}$ (HR), $ROI_{thermal}$ (BT), $ROI_{AT}$ (RR), and $ROI_{neck}$ (for truncus motion determination) are the regions of interest corresponding to face (wide), face (narrow), anterior thorax, and neck, respectively, as shown in Figure 3. Using a stereo depth camera, the facial area was determined using the bounding box algorithm *via* OpenCV library and the average human facial dimension (Zhuang et al., 2010). For extracted facial area, an RGB camera was adopted to determine facial landmarks A to D [Figure 3 (B; Chart 2)]. $ROI_{BVP}$ was determined using A to C, and $ROI_{neck}$ (3 × 3 cm) was placed below point D corresponding to the jaw. $ROI_{thermal}$ (5 × 7 cm) was placed 3 cm above B. Using typical human facial dimensions, $ROI_{AT}$ [20 (H) cm ×18(W) cm] was fixed 12 cm below D (Bellemare et al., 2003).

#### Heart Rate Estimation

The VISC-Camera determines HR by sensing facial skin tone changes induced by an arterial pulsation called blood volume pulse (BVP) using an RGB image-based remote photoplethysmography (rPPG) method (Figure 3B). To extract the BVP signal, the VISC-Camera detects the human face using the Haar cascade classifier from the OpenCV library (Viola and Jones, 2004). The median flow object tracking algorithm was adopted from the OpenCV library to track facial ROI for HR measurement without being affected by body motion, including headshake (Kalal et al., 2010). To efficiently acquire the BVP signal, we selected $ROI_{BVP}$ based on the human facial arterial anatomy using facial landmark points A–C [Figure 3 (B; Chart 2)] (von Arx et al., 2018). The landmark points are determined by a neural network-based facial landmark detection algorithm from the DLIB library (King, 2009). The BVP signal is extracted from the spatial average of the RGB green color signal of the ROI via multiresolution analysis of the maximal overlap discrete wavelet transform (MODWTMRA) with order four Symlet wavelet filter, decomposition level 4, and bandpass filter (0.7–2.5 Hz). To avoid aperiodic waves, we adopted the autocorrelation function (ACF) on the extracted BVP. Finally, HR is estimated using the root multiple signal classification (MUSIC) algorithm with elements of one at a sampling rate of 30 Hz (Barabell, 1983).

#### The Truncus Motion Removal Algorithm for Respiratory Rate Estimation

The VISC-Camera determines RR by monitoring the examinee’s volume of the anterior thorax [ $V_{AT}\left(t\right)$ ] using a stereo depth camera. The truncus motion removal algorithm (TMRA) enables accurate RR measurement from the respiratory anterior thoracic motion by extracting non-respiratory front-back truncus movements determined by neck motions (Figure 1). Neck as ROI was adopted to estimate non-respiratory front-back motion because there are no respiratory muscles on the neck. $V_{AT}\left(t\right)$ is determined as follows:

$$\begin{array}{c} V_{AT}\mathrm{(t)=}\int_{0}^{t}\sum_{\mathrm{x=1}}^{\mathrm{424}}\sum_{\mathrm{y=1}}^{\mathrm{240}}\left(S_{\mathrm{pixel}}\mathrm{(t)\times}\frac{\left(\Delta Z_{\mathrm{AT,xy}}\left(t\right)\mathrm{-\Delta}Z_{\mathrm{truncus}}\left(t\right)\right)}{\mathrm{\Delta t}}\mathrm{\times RO}I_{\mathrm{xy}}\right)\mathrm{dt} \end{array}$$ (2)

$$\mathrm{RO}I_{\mathrm{xy}}=\{\begin{array}{cc} \mathrm{1,} & \mathrm{if}\left(\mathrm{x,y}\right)\mathrm{\in RO}I_{\mathrm{AT}}\\ \mathrm{0,} & \mathrm{otherwise} \end{array}$$

where $Z_{AT,xy}\left(t\right)\;$ are the distances from VISC-Camera to an anterior thorax point (x,y) , $Z_{truncus}\;$ is the truncus distance determined as the average distance of the $ROI_{neck}$ from VISC-Camera, 424 and 240 are the maximum pixel numbers of the stereo depth camera corresponding to horizontal and vertical directions, respectively. Δt is a sampling interval (33 ms). $S_{pixel}\left(t\right)$ is the area of a pixel at a distance of $Z_{truncus}\left(t\right)$ as shown below, 86° and 57° are the stereo depth camera’s horizontal and vertical field of view angles, respectively.

$$\begin{array}{c} S_{\mathrm{pixel}}\left(t\right)=\frac{2\times \left(Z_{\mathrm{truncus}}\left(t\right)\right)\times \tan\left(\frac{\mathrm{86}{}^{\circ}}{2}\right)}{\mathrm{424}}\times \frac{2\times \left(Z_{\mathrm{truncus}}\left(t\right)\right)\times \tan\left(\frac{\mathrm{57}{}^{\circ}}{2}\right)}{\mathrm{240}} \end{array}$$ (3)

The facial landmark point D, which was coordinated in the HR estimation process, determines the location of the $ROI_{neck}$ and the $ROI_{AT}$ to reduce computational load [Figure 3 (A; Chart 2)]. In addition, a bandpass filter (0.1–0.6 Hz) excludes higher frequency artifacts induced by the examiner’s handshake. To estimate RR from a short-time signal, we used ACF to find the periodicity of the respiratory signal and root MUSIC algorithm (with elements of one at a sampling rate of 30 Hz) for RR determination.

#### Body Temperature Estimation

The VISC-Camera estimates BT from the $ROI_{thermal}$ using a thermal camera (Figure 3C). The $ROI_{thermal}$ is located 3 cm above the facial landmark point B [Figure 3 (C; Chart 2)]. We conducted linear regression analysis to estimate BT from the facial temperature ( $T_{face}$ ). The linear equation is expressed as follows:

$$\begin{array}{c} \mathrm{BT=A\times}T_{\mathrm{face}}\mathrm{+B} \end{array}$$ (4)

where $\;\;T_{face}$ is an average temperature within the $ROI_{thermal}$ . We excluded pixels indicating temperatures below 34°C or above 42°C in the $ROI_{thermal}$ . A and B are regression coefficients.

#### Image Quality Assessment

Taking stable images from a proper position is essential in measuring reliable signals using image-based remote methods. Therefore, the VISC-Camera screening is according to the following procedures. First, with 3D visual assistance on the screen, the examiner can orientate the device to the examinee. Next, to begin measurement, the VISC-Camera assesses the examinee’s posture with respect to angles, i.e., roll and yaw, using facial landmark points A-C. After measurement starts, the VISC-Camera detects sudden motions using a second derivative-based blur detection method while capturing images (Pech-Pacheco et al., 2000). We used the Laplacian operator as a second derivative operator with the following 3 × 3 kernel:

$$\begin{array}{c} \left[\begin{array}{ccc} 0 & 1 & 0\\ 1 & \mathrm{-4} & 1\\ 0 & 1 & 0 \end{array}\right] \end{array}$$ (5)

The pre-defined motion detection threshold value of the variance of the absolute value was Laplacian_Variance<100 .

### Clinical Testing of VISC-Camera at the First Central Hospital of Mongolia for COVID-19 Patients

We conducted clinical testing of the VISC-Camera in 154 patients who tested positive for COVID-19 according to RT-PCR (aged 18–81 years; 87 males, 67 females) within the initial 48 h of hospitalization at the First Central Hospital of Mongolia. A control set comprised 147 healthy volunteers (aged 18–85 years; 70 males, 77 females). All patients were afebrile (BT < 37.5°C) following administration of antiviral agents (umifenovir 200 mg/day or favipiravir 200 mg/day). Febrile patients in the intensive-care unit were not included. All healthy volunteers were examined using COVID-19 symptoms-based questionnaires and showed no symptoms for 1 week before and after VISC-Camera screening. We did not use criteria in body temperature, which excludes an examinee with and without COVID-19. A summary of the demographic of the participants is shown in Table 1. Figure 1 shows the VISC-Camera measurement setup in the standing position. We did not give any instructions on posture to examinees. Standing, sitting, and supine examinees are included in our study. There are possible effects of posture on RR and HR; however, the RR of COVID-19 patients drastically increased compared to healthy volunteers. The clinical tests were conducted indoors with moderate illumination levels (600–1,100 lux). For reference, a respiratory belt, visual respiratory counting, a fingertip pulsimeter, and a thermometer were used. We used a respiratory belt and visual respiratory counting as references of RR for healthy volunteers and COVID-19 patients, respectively. Because person-to-person contact was strictly limited in the isolation unit of COVID-19. Prior to a clinical study, we tested the concordance between respiratory belt-derived RR and that determined by visual respiratory counting. In order to evaluate false positive cases induced by another infectious disease with systemic inflammation, we recruited 33 pediatric pneumonia inpatients (aged 1–14 years; 18 males, 15 females) measured by the VISC-Camera at the Central Hospital of Songinokhairkhan District in Mongolia. This study was approved by the Tokyo Metropolitan University ethics committee (approval number H20-038). All participants gave written informed consent. We obtained the photograph licensing of Figure 1 from the patient.

**TABLE 1:** Demographics of the patients with COVID-19 and the healthy volunteers.

	COVID-19	Healthy	*p*-value
Heart rate	76.4 ± 13.2	75.7 ± 10.9	0.60
Respiratory rate	22.1 ± 2.7	15.1 ± 2.4	<0.01
Body temperature	36.41 ± 0.20	36.37 ± 0.22	0.12
Logit score	1.8 ± 1.4	-1.7 ± 1.2	<0.01
Age	44.8 ± 14.8	44.7 ± 14.1	0.93
Sex (male/female)	87/67	70/77	0.12

### Statistical Analysis

Pearson’s correlation coefficient and Bland–Altman plots were used to analyze the correlation between the measurement of the VISC-Camera and the references. The LRA classification model results were used to calculate the sensitivity, specificity, negative predictive value (NPV), and positive predictive value (PPV). A *t*-test was conducted to statistically assess the vital signs’ mean rate of the two groups. Ten-fold cross-validation was performed, and the receiver operating characteristic curve was calculated to evaluate the accuracy of LRA models.

## Results

**FIGURE 4:**
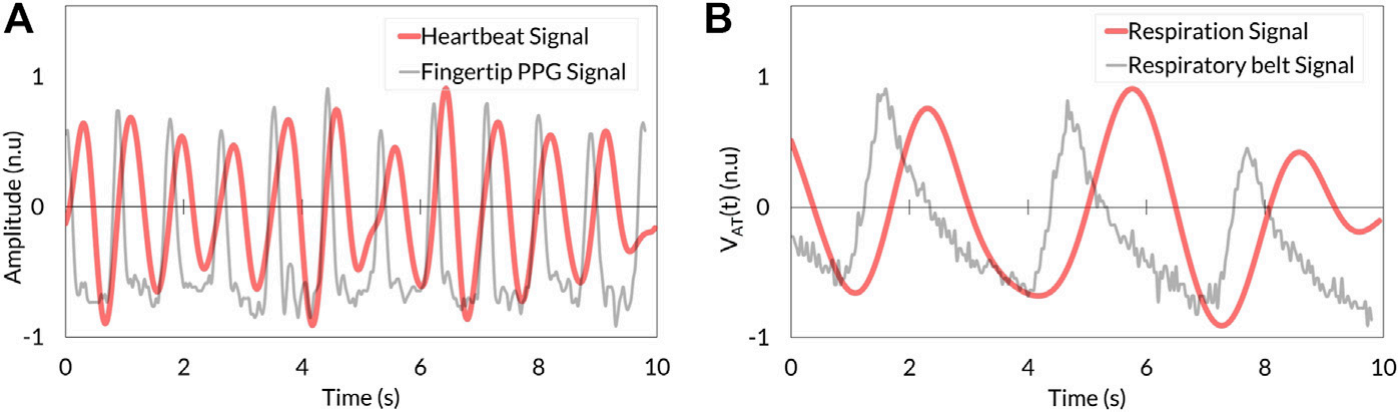
Comparison between VISC-Camera-derived vital signals **(red)** and references **(grey)**. **(A)** Heartbeat signal. **(B)** Respiration signal. PPG, photoplethysmography.

The heartbeat signal measured by the RGB camera showed similar periodic fluctuation to that determined by fingertip photoplethysmography (Figure 4A). Respiration signal determined by stereo depth camera indicated cyclic oscillation same as that derived by the respiratory belt (Figure 4B).

**FIGURE 5:**
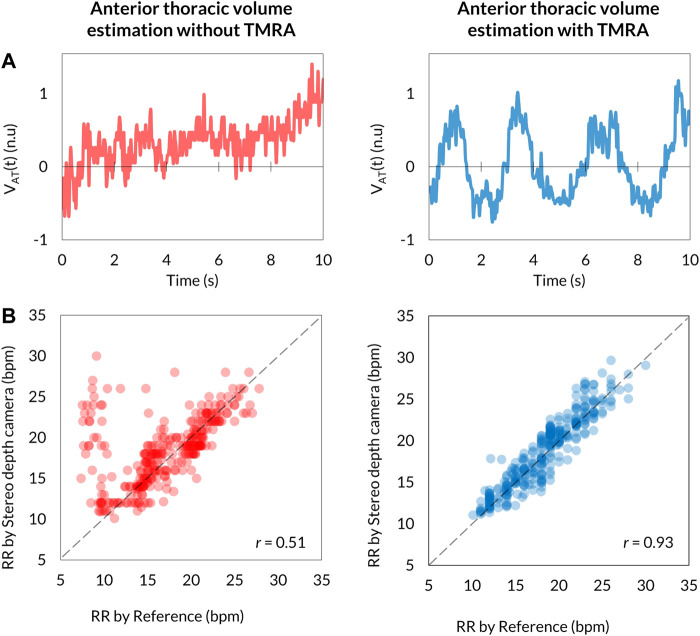
Comparison of $V_{AT}\left(t\right)$ measurements between without (left) and with (right) TMRA. **(A)** Respiration signals (left; without TMRA, right; with TMRA). **(B)** Scatter plots of correlation between the Stereo depth camera-determined RR and the reference value (left; without TMRA, *r* = 0.51, right; with TMRA, *r* = 0.93).

Respiratory signals determined as $V_{AT}\left(t\right)$ with (right) and without (left) TMRA are shown in Figure 5. TMRA drastically improved the cyclic feature of the respiratory signal (Figure 5A). TMRA greatly increased the correlation coefficient between RR determined by the reference and VISC-camera-derived RR from 0.51 (Figure 5B; left; without TMRA) to 0.93 (Figure 5B; right; with TMRA).

BT, defined as the same as the axillary temperature, is determined using thermal camera-monitoring of the temperature ( $T_{face}$ ) of the facial region of interest (ROI; Figure 2C) using the following equation:

$$\mathrm{BT}=\mathrm{0.78}\times T_{\mathrm{face}}+\mathrm{8.99}$$

**FIGURE 6:**
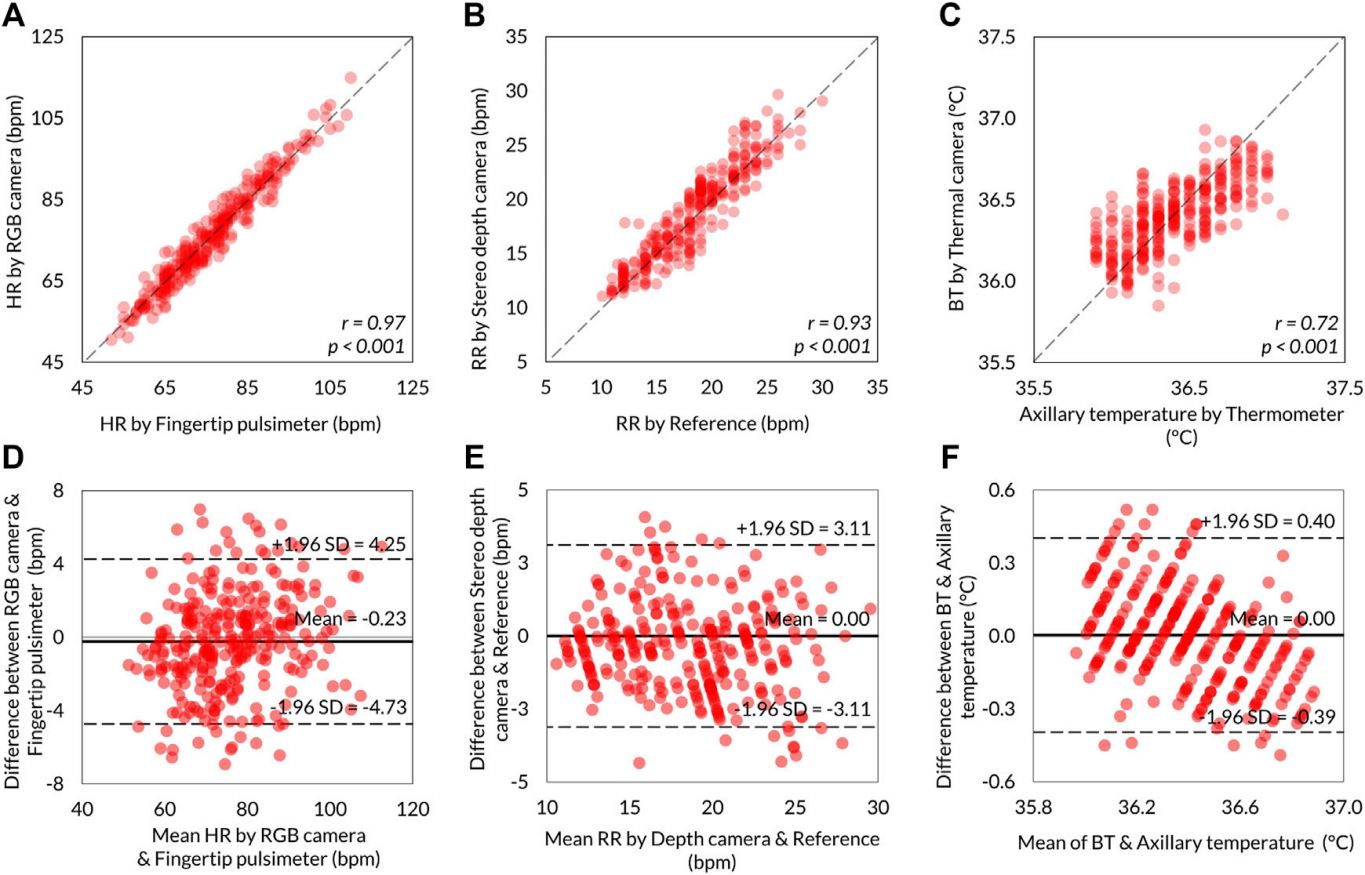
Summary of the VISC-Camera’s vital signs measurement accuracy (n = 301). Scatter plots showing the relationship between VISC-Camera determined vital signs and reference vital sign values. **(A)** HR with *r* = 0.97, *p* < 0.001, RMSE = 2.7. **(B)** RR with *r* = 0.93, *p* < 0.001, RMSE = 1.6. **(C)** BT with *r* = 0.72, *p* < 0.001, RMSE = 0.2. The Bland–Altman plots illustrating 95% limits of agreements of RR, HR, and BT measurements by the VISC-Camera and the references. **(D)** For HR, the RGB camera and fingertip pulsimeter ranged from −4.7 to 4.2 bpm vs. mean (σ = 2.3). **(E)** For RR, the stereo depth camera and the reference ranged from −3.1 to 3.1 bpm vs. mean (σ = 1.6). **(F)** BT and axillary temperature ranged from −0.39 to 0.40°C vs. mean (σ = 0.2).

The level of agreements between the VISC-Camera and the references were assessed using the Pearson correlation coefficient (*n* = 301) and the Bland–Altman plot. Correlation scatter plots for HR, RR, and BT are shown in Figures 6A,B,C, respectively. VISC-Camera-determined HR, RR, and BT significantly correlated with those measured using references (HR: *r* = 0.97, *p* < 0.001; RR: *r* = 0.93, *p* < 0.001; BT: *r* = 0.72, *p* < 0.001). The root mean squared errors of HR, RR, and BT were 2.7, 1.6, and 0.2, respectively. Figures 6D,E,F show the Bland–Altman plots for HR, RR, and BT determined by the VISC-Camera and the references. The 95% limits of agreement of HR, RR, and BT measurements ranged from −4.7 to 4.2 bpm (*σ* = 2.3), -3.1 to 3.1 bpm (*σ* = 1.6), and -0.39 to 0.40°C (*σ* = 0.2), respectively.

**FIGURE 7:**
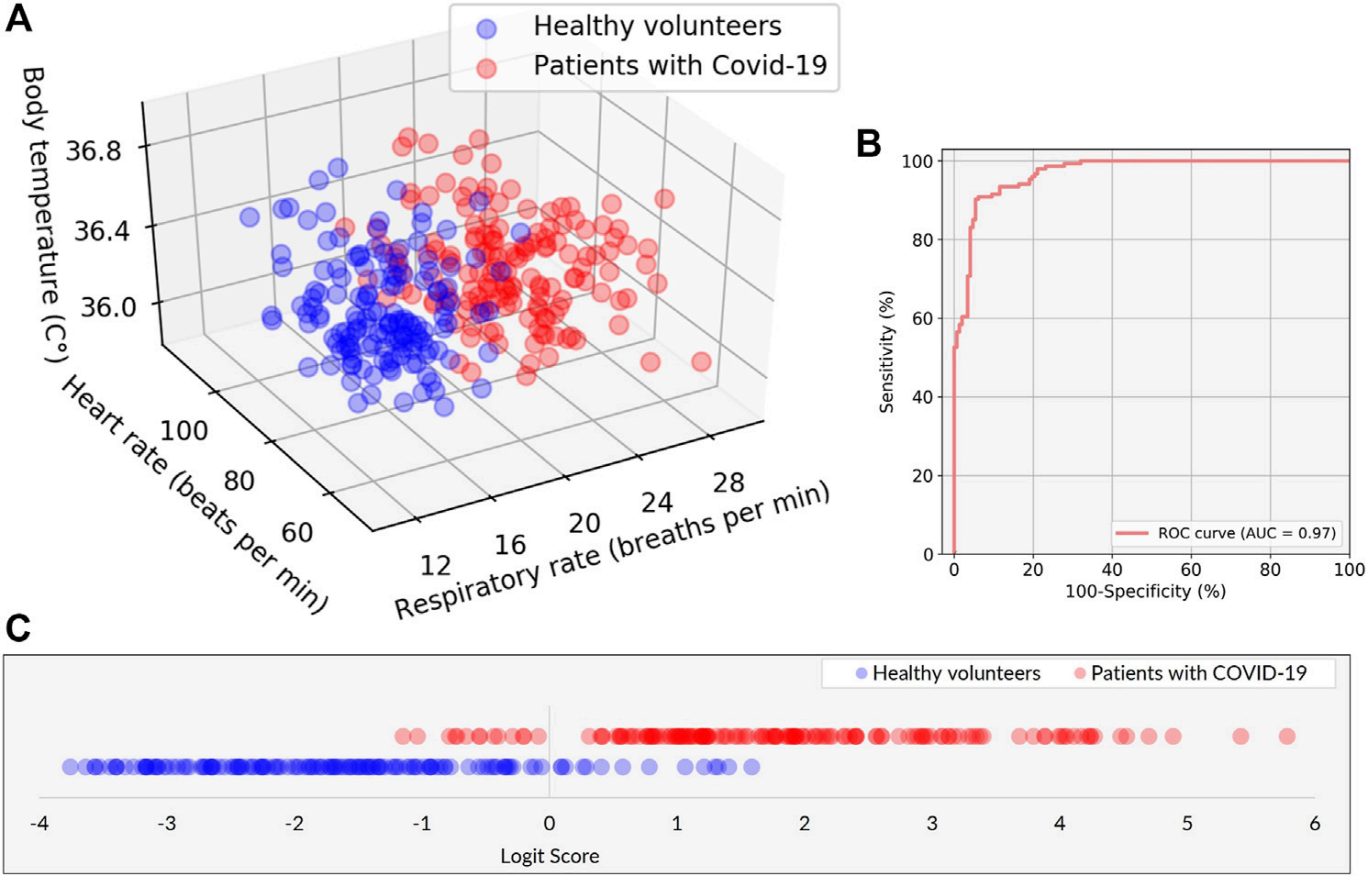
Performance of the classification model in discriminating between patients with COVID-19 and healthy volunteers. **(A)** Scatter plots of the three explanatory variables of RR, HR, and BT in patients with COVID-19 (red) and healthy volunteers (blue). **(B)** The receiver operating characteristic (ROC) curve with area under the curve (AUC) of 0.97. **(C)** The classification result is represented by the logit scores (Logit Score ≥0 ⇒Suspected as COVID-19, Logit Score <0 ⇒Healthy).

The 3D plot of RR, HR, and BT determined by the VISC-Camera is shown in Figure 7A. Although all patients with COVID-19 were afebrile, the 3D plot shows two groups: the patient and the healthy volunteer groups. This is because the RRs of infected patients were significantly higher than those of healthy volunteers (patients with COVID-19: mean RR = 22.1 bpm (*σ* = 2.7); healthy volunteers: mean RR = 15.1 bpm (*σ* = 2.4); two-tailed *p* < 0.05). To separate patients from healthy volunteers more accurately, the following logit regression analysis (LRA) equation was determined by adopting RR, HR, and BT as explanatory variables:

$$\begin{array}{c} \mathrm{Logit Score=log}\frac{p}{\mathrm{1-p}}\mathrm{=-9}\mathrm{.192+0.505\times}\mathrm{RR-}\mathrm{0.006\times}\mathrm{HR+}\mathrm{0.0101\times}\mathrm{BT}\\ \begin{array}{l} \mathrm{Logit Score\geq 0 \Rightarrow Suspected as COVID-19}\\ \mathrm{Logit Score<0 \Rightarrow Healthy} \end{array} \end{array}$$

where p is the probability, $\frac{p}{1-p}$ is the corresponding odds. In the screening of afebrile patients with COVID-19, a 10-fold cross-validation revealed 91% sensitivity, 90% specificity, 91% positive predictive value (PPV), and 90% negative predictive value (NPV) (Figure 7C). From the receiver operating characteristic (ROC) curve, the LRA model achieved an area under curve (AUC) of 0.97 (Figure 7B). Also, to evaluate false positive cases induced by another infectious disease with systemic inflammation, we tested our LRA model by adding 33 recruited pediatric pneumonia patients to 154 patients with COVID-19 and 147 healthy volunteers. The result revealed 91% sensitivity, 74% specificity, 75% PPV, and 90% NPV. Even the false positive rate increased; however, the most important parameters in quarantine screening, i.e., sensitivity and NPV, did not change.

## Discussions

The VISC-Camera can be used as an Internet of Things device for an infectious disease surveillance platform, as proposed in our previous study (Sun et al., 2020). It can also be used routinely in hospitals as a daily vital signs recording tool because it determines RR, HR, and BT within 10 s and correlates significantly with reference values. Generally, healthcare professionals take more than 3 min to measure and record vital signs. Therefore, in addition to screening, the proposed system may reduce the everyday burden on medical staff responsible for the care of patients with COVID-19.

In addition, sequential organ failure assessment (SOFA) has long been used to predict ICU mortality. However, in recent years, and in particular, following the publication of the Sepsis-3 guidelines (Singer et al., 2016), quick SOFA (qSOFA) has been more commonly used in clinical practice than SIRS or SOFA scores, which are better suited to evaluation during the early stages of sepsis screening. The qSOFA approach is also used when a sudden change in patient status is suspected. Although qSOFA uses only three parameters (RR, blood pressure, and consciousness), the traditional RR measurement is relatively laborious for medical staff. Therefore, a device to measure RR quickly and easily could be a helpful tool in an emergency clinical setting.

Although limited to supine position, Dong et al. recently reported non-contact COVID-19 screening using continuous-wave radar-based non-contact sleep monitoring equipment via XGBoost and LRA model (Dong et al., 2022). However, the VISC-Camera enables non-contact COVID-19 screening in standing and sitting postures, as well as in supine positions. In addition, our study focuses on not only technical backgrounds but also clinical findings, such as the success of non-febrile COVID-19 patients screening by non-contact vital signs monitoring.

In our study, the patients were treated with antiviral medication in advance; thus, their conditions differed from the initial characteristics. Despite their lack of fever, we found a drastic increase in RR in patients with COVID-19 infection. Therefore, the ability of the proposed system to accurately measure RR during preliminary screening may contribute to the reduction of false-negative test results for COVID-19 infection.

Our study has some limitations. Critically ill patients with COVID-19 were excluded, and all participants were recruited from one country. Therefore, further data are needed that encompass examinees with various degrees of COVID-19 severity, diverse ethnicities from various countries, and a wide range of ages to increase the general-purpose versatility of the VISC-Camera. In this study, the VISC-Camera was used in air-conditioned areas with moderate illumination levels (600–1,100 lux). Therefore, the VISC-Camera should also be tested in other, less optimal environments. The VISC-Camera detects COVID-19-induced inflammatory responses rather than the SARS-CoV-2 virus itself. Therefore, there are possibilities to misdiagnose the other infectious diseases as COVID-19, such as pneumonia or influenza. The VISC-Camera was developed for preliminary screening and not to define the diagnosis.

The portable VISC-Camera described here, which conducts infection screening within 10 s in a no-contact manner, appears promising for the preliminary screening of potential COVID-19 infection in afebrile patients.

## Data Availability

The raw data supporting the conclusion of this article will be made available by the authors, without undue reservation.

## Supplementary Material

Supplementary Video S1 A demonstration video of the VISC-Camera’s vital signs-related signals extraction procedure.
